# Left Ventricular Non-compaction Cardiomyopathy: Delayed Diagnosis and Deleterious Outcomes

**DOI:** 10.7759/cureus.16041

**Published:** 2021-06-29

**Authors:** Steven Hamilton, Shenae Cummings, Samir Shah

**Affiliations:** 1 Internal Medicine, Jersey Shore University Medical Center/Saint Francis Medical Center Program, Trenton, USA; 2 Internal Medicine, Heart Institute of the Caribbean, Kingston, JAM; 3 Cardiology, Saint Francis Medical Center, Trenton, USA; 4 Cardiology, Jefferson University Hospital, Philadelphia, USA

**Keywords:** heart failure with reduced ejection fraction, delayed diagnosis, cardiomyopathy, non-compaction, cardiac mri

## Abstract

Cardiomyopathy and associated heart failure have uncommon etiologies, which when diagnosed reduce patients’ morbidity and mortality. One such entity is left ventricular non-compaction cardiomyopathy (LVNC). Still, a relatively uncommon entity, the manifestation of LVNC may range from asymptomatic to left ventricular dysfunction, congestive heart failure, ventricular tachycardia, sudden cardiac death, and thromboembolic complications. If not pursued as a possible etiology of non-ischemic cardiomyopathy, patients may have significantly increased morbidity prior to eventual diagnosis. Patients are often predisposed to ventricular arrhythmias requiring implantable cardiac defibrillator placement. Additionally, due to the depth of trabeculations, there is an associated thromboembolic risk requiring therapeutic anticoagulation. We present the case of a 41-year-old man with progressively worsening heart failure due to undiagnosed LVNC and the associated deleterious manifestations and outcomes.

## Introduction

Left ventricular non-compaction cardiomyopathy (LVNC) is the most recent cardiomyopathy classified as a distinct entity by the American Heart Association and remains a heterogeneous and complex entity. Still a relatively uncommon entity, LVNC may go undetected with deleterious outcomes for the affected individual. Establishing the diagnosis of LVNC requires a high index of clinical suspicion when a patient presents with non-ischemic cardiomyopathy. The diagnosis of LVNC carries with it an increased risk of ventricular arrhythmias, systemic embolism, progressive heart failure and even sudden cardiac death [1]. LVNC portends an increased risk for poor outcomes and should be detected and treated both aggressively and appropriately.

## Case presentation

**Figure 1:**
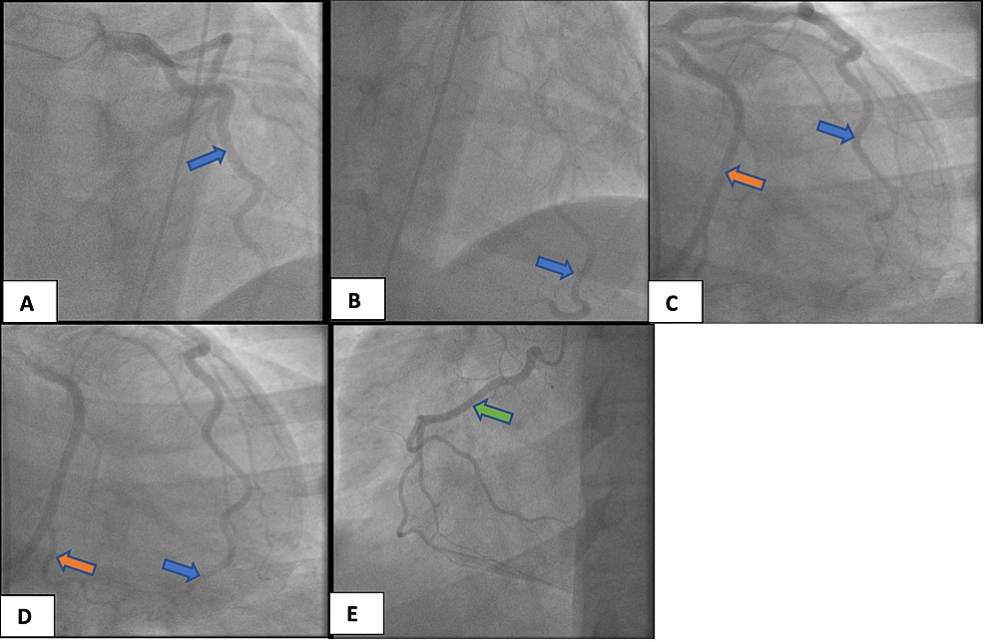
Left heart catheterization with no significant coronary artery disease. (A) Left anterior descending artery (LAD, blue arrow). (B) Distal LAD (blue arrow). (C) LAD (blue arrow) and left circumflex artery (LCx, orange arrow). (D) Distal LAD (blue arrow) and distal LCx (orange arrow). (E) Right coronary artery (RCA, green arrow).

**Figure 2:**
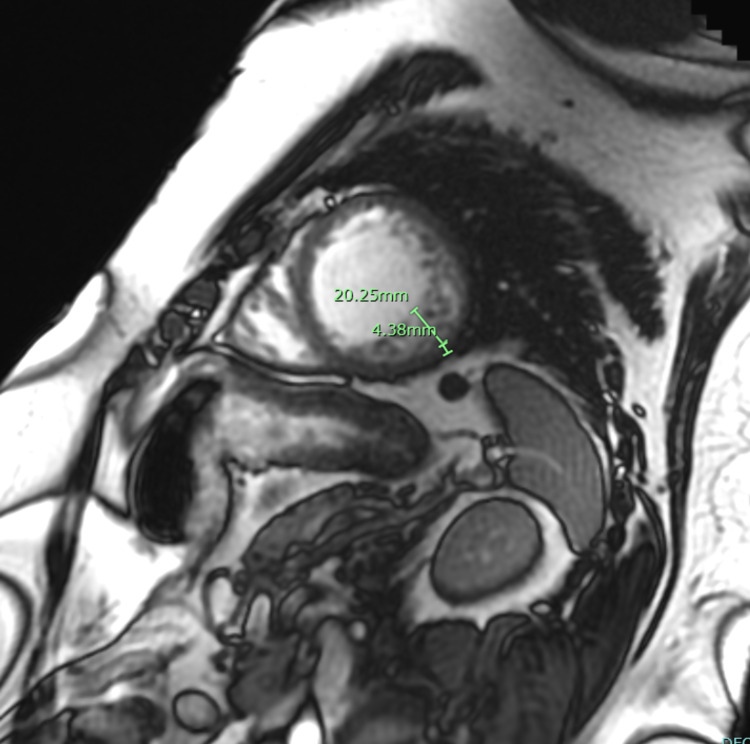
Cardiac MRI revealed in the inferolateral area, mid left ventricle the non-contrast fraction measuring approximately 20mm and the underlying compacted left ventricular wall measures 4mm. There is apical thinning and highly trabeculated.

We present a 41-year-old man with a past medical history of chronic New York Heart Association (NYHA) Class II-III heart failure with reduced ejection fraction (HFrEF), non-ischemic cardiomyopathy, obstructive sleep apnea, chronic kidney disease and gastroesophageal reflux disease. His home medications were Entresto 24/26mg orally twice daily, carvedilol 3.125mg orally twice daily, pantoprazole 40mg orally twice daily and Maalox 30cc orally every six hours as needed for indigestion. There was no known family history of heart disease, sudden or premature death. He is a former smoker and does use alcohol or illicit drugs, and had serum ethanol less than 10 and urine drug screen negative. He was initially diagnosed with cardiomyopathy of uncertain etiology, HFrEF and he was told that his ejection fraction (EF) was 20%-25% (no records available), two years prior at another facility. There was no ischemic workup at that time and he was discharged on carvedilol, furosemide, lisinopril and metoprolol tartrate (unknown dosages of medications). Subsequently, he had his first presentation to our institution with exertional dyspnea in the context of decompensated HFrEF. At that time, he underwent cardiac catheterization which revealed no significant coronary artery disease (Figures 1A-1E). Thereafter, he was discharged on low-dose carvedilol, as needed torsemide and Entresto. Aldactone was withheld due to hypotensive episodes. He was advised to have a repeat transthoracic echocardiogram in three months to assess left ventricular systolic function on optimized medical therapy but this was not done. Additionally, he was recommended for outpatient cardiac magnetic resonance imaging to delineate a possible etiology for his nonischemic cardiomyopathy. Seven months later, he underwent cardiac magnetic resonance imaging, which showed a non-compacted (NC) to compacted myocardium ratio of greater than 2.3 (Figure 2).

 

**Figure 3:**
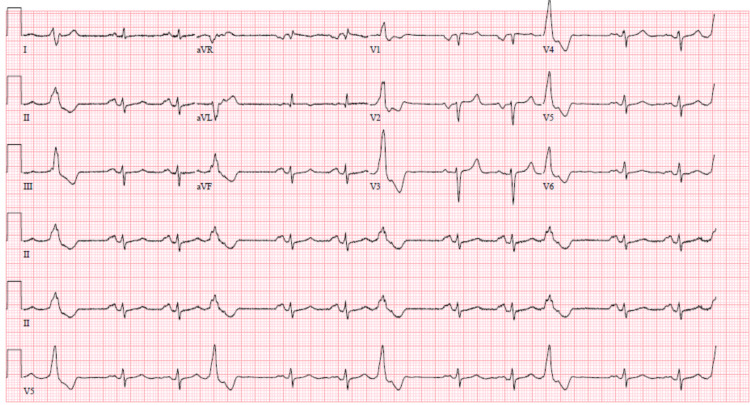
EKG with normal sinus rhythm at 73 beats per minute, ventricular trigeminy, no pathologic Q-waves, left axis deviation, possible left atrial enlargement, poor precordial R-wave progression and nonspecific T-wave abnormality.

Thereafter, he re-presented with two weeks of worsening dyspnea on exertion, bilateral lower extremity swelling and significant weight gain. On presentation, vital signs were as follows, blood pressure 125/80mmHg (mean arterial pressure of 88mmHg), regular heart rate of 76 beats per minute with increased pulse volume, respiratory rate 24 breaths per minute, temperature 98.2F and SpO_2_ 99% on ambient air. His normal dry weight was approximately 330lbs (149.7kg) and his weight at the time of presentation was 350lbs (158.8kg). Physical examination revealed reduced breath sounds bilaterally with significant rales, along with bilateral lower extremity pitting edema. The remainder of his systems examination was within normal limits. EKG demonstrated normal sinus rhythm at 73 beats per minute, ventricular trigeminy, no pathologic Q-waves, left axis deviation, possible left atrial enlargement, poor precordial R-wave progression and nonspecific T-wave abnormality (Figure 3). Subsequent transthoracic echocardiogram with definity echo contrast revealed severe LV systolic dysfunction, EF 25%-30% (Video 1). 

**Video 1:** Transthoracic echocardiogram with definity contrast which revealed severe global LV systolic dysfunction, ejection fraction 25%-30%. LV - left ventricular

**Figure 4:**
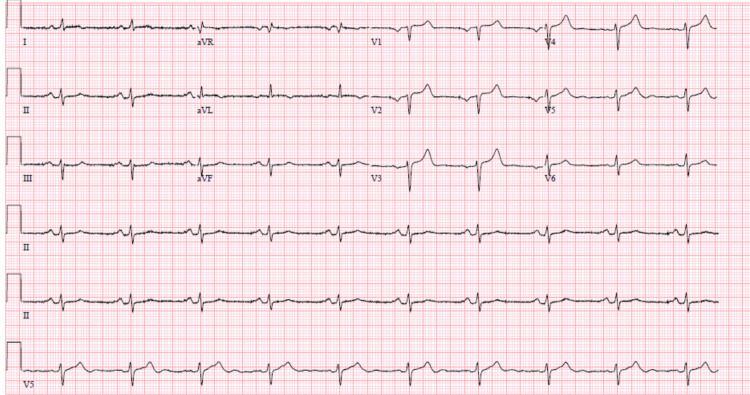
EKG post dual-chamber AICD with atrial-paced rhythm, prolonged AV conduction, left axis deviation. AICD - automated implantable cardiac defibrillator; AV - atrioventricular

The patient presented with clinical features consistent with decompensated heart failure. As such, he was diuresed successfully to euvolemia with intravenous furosemide. He experienced persistent sinus bradycardia with a nadir of 40 beats per minute likely of multifactorial origin - deoxygenation from congestive heart failure [2], superimposed on obesity hypoventilation and inadequately treated obstructive sleep apnea. Even after aggressive diuresis and improved oxygenation with the utilization of continuous positive airway pressure therapy his bradycardia persisted. Our thought process was that the patient would not be able to tolerate maintenance beta-blockade (necessary guideline-directed medical therapy [GDMT] for his heart failure) given his persistent nocturnal sinus bradycardia into the 40s. Concurrently he had symptomatic salvos of nonsustained ventricular tachycardia. In this setting and combined with the fact that he had been on GDMT, he received a dual-chamber cardiac defibrillator system (ICD). Ultimately, we opted for implantation of a dual-chamber cardiac defibrillator system in the setting of sinus node dysfunction, as he benefited from atrial pacing capability to allow better titration of his carvedilol. Repeat EKG post ICD implantation revealed an atrial-paced rhythm (Figure 4). Given his diagnosis of non-compaction and deep trabeculations he was continued on therapeutic anticoagulation. However, there was mild hypotension limiting the intensification of GDMT. He was discharged on torsemide, carvedilol, apixaban, low-dose Entresto and spironolactone in a stable condition.

At two months post dual-chamber cardiac defibrillator system placement and GDMT, the patient was asymptomatic and euvolemic with adequate perfusion. However, there was persistent hypotension limiting the use of afterload reducing agents and this will be followed closely in the outpatient setting. On discharge, we recommended that his first-degree relatives, his children, be screened. Additionally, his outpatient cardiologist will arrange for potential genetic testing of the patient.

## Discussion

LVNC is a heterogeneous and complex entity. While the American Heart Association includes it among genetic cardiomyopathies, the European Society of Cardiology treats it as an unclassified cardiomyopathy. It may present in a sporadic or familial form, isolated or associated with other heart diseases, affecting only the left ventricle or both and can sometimes appear as a mixed phenotype in patients with other cardiomyopathies. Different forms of clinical presentation are also associated with its different morphological manifestations, and non-compaction of the left ventricle may be triggered by other physiological or pathological processes [3]. Non-compacted myocardium (NCM) was first reported by Grant in 1926 as a heterogeneous myocardial disorder characterized by prominent ventricular trabeculation, intertrabecular recesses, and bilayered myocardium composed by a compacted and an NC layer [4,5]. The etiology of LV non-compaction is uncertain, and several etiological bases have been implicated. It is believed to be due to pathogenic mechanisms resulting in a failure in the final phase of myocardial morphogenesis, or myocardial compaction. Additionally, increasing evidence has supported a genetic basis by identifying mutations in the genes that encode sarcomeric, cytoskeletal and nuclear membrane proteins. Though, traditionally, it is considered to be the result of an interruption of normal myocardial development between weeks 5 and 8 of embryogenesis [6].

Classically, LVNC has been referred to as the triad of heart failure, arrhythmias and embolic episodes. However, its presentation is very heterogeneous and covers a broad spectrum, from asymptomatic patients diagnosed by familial or another screening context to advanced heart failure and sudden death [3]. With respect to diagnosis, LVNC is often overlooked and it is likely that many cases are undiagnosed. An initial challenge that exists is that non-compaction is most prominent at the apex of the left ventricle, which is known to be difficult to visualize on an echocardiogram. The enhancement of endomyocardial definition assists in overcoming this diagnostic limitation [7]. However, in our patient's case, a transthoracic echocardiogram with definity contrast did not reveal non-compaction. The accepted echocardiographic diagnostic criteria by Jenni et al. are (1) the absence of coexisting cardiac anomalies; (2) the presence of multiple, prominent trabeculations; (3) multiple deep intertrabecular recesses filled with blood from the ventricular cavity, as demonstrated by colour Doppler imaging; and (4) the maximal end-systolic ratio of NC endocardial layer to the compacted myocardium (C) of NC to C ratio >2 [8]. These echocardiographic criteria were not met for our patient.

The outpatient cardiac MRI was done shortly prior to presentation demonstrated an NC to C ratio of >2.3 in end-diastole, estimated left ventricular EF (LVEF 33%). This met the criteria validated by Petersen et al., with values for sensitivity, specificity, and positive and negative predictions of 86%, 99%, 75%, and 99%, respectively [9]. The Petersen criteria are a ratio of NC to C >2.3, with an acquisition in end-diastole. A primary advantage of cardiac MRI is the 3D configuration, which allows imaging of the entire volume of the heart with lower investigator dependency and without limitations caused by a patient’s constitution [10]. The cardiac MRI outperforms echocardiography in identifying myocardial trabeculations, leading to increased on-target detection of the NCM. Additionally, cardiac MRI offers a reliable assessment of the left ventricular function. Limitations with respect to cardiac MRI included lack of availability in our community hospital setting and increased cost. One technical limitation is the use of black blood imaging, which was done in this case. There may be stagnant blood between myocardial trabeculations causing pseudothickening of the ventricular wall, leading to either misdiagnosis or overlooking of LVNC.

Currently, there are no established guidelines by which to treat patients with LVNC. Our patient was treated similarly to a typical patient with LVNC with some modifications. The heart failure component was treated with GDMT, which included a beta-blockade, neprilysin/angiotensin receptor blocker and aldosterone antagonist. Ventricular tachyarrhythmia was addressed with an implantable cardiac defibrillator. Our thought process was as follows. If his EF remained less than 35% then he would require an automated implantable cardiac defibrillator (AICD) for primary prevention. If despite this his bradycardia persisted, we would need to consider biventricular pacemaker placement/cardiac resynchronization therapy in addition to his AICD (he would likely require greater than 40% pacing while being maintained on carvedilol). Indications for the dual-chamber cardiac defibrillator system were non-ischemic cardiomyopathy (LVNC), NYHA Class II-III CHF (despite optimal medical therapy >90 days) and sinus node dysfunction limiting the required beta-blocker for HFrEF management.

Additionally, due to the depth of trabeculations and the associated thromboembolic risk, he was continued on therapeutic anticoagulation. Patients require close and vigilant monitoring as there is increased associated morbidity and mortality. Current guidelines recommended that specific genetic testing be conducted for family members and appropriate relatives following the identification of an LV non-compaction causative mutation in the index case.

## Conclusions

Non-compaction cardiomyopathy should be considered when patients present with non-ischemic cardiomyopathy. It is important to pursue a diagnostic workup for a potential etiology of this non-ischemic cardiomyopathy. Patients with LVNC often present with heart failure and are treated the same as typical heart failure with GDMT. They are predisposed to ventricular tachycardia, possibly manifesting as syncope and as a result, they typically receive an implantable cardiac defibrillator. Due to the depth of trabeculations and increased thromboembolic risk they should be on therapeutic anticoagulation. With respect to wider impact, LVNC confers increased morbidity and mortality not only for the patient but also potentially for family members.
